# Gynecological laparoscopic surgery in the shade of COVID-19 pandemic

**DOI:** 10.3906/sag-2004-272

**Published:** 2020-06-23

**Authors:** Şadıman KIYKAÇ ALTINBAŞ, Ömer Lütfi TAPISIZ, Yaprak ENGİN ÜSTÜN

**Affiliations:** 1 University of Health Sciences, Etlik Zübeyde Hanım Women’s Health Training and Research Hospital, Ankara Turkey

**Keywords:** COVID-19, SARS-CoV-2, pandemic, laparoscopy

## Abstract

A global public health problem with a high rate spread and transmission, Coronavirus outbreak has become the most talked-about matter throughout the world. We are severely affected by the nations with vast numbers of deaths; it was hard to predict such a colossal pandemic with terrifying consequences. Elective surgeries are limited, but situations requiring an urgent gynaecological or obstetric surgical approach must still be performed during the COVID-19 pandemic. Concerns regarding surgical safety and the risk of viral transmission during surgery are of great importance. In this review, we aimed to summarize the concepts related to laparoscopic gynecological surgery during COVID-19 pandemic in the light of current literature.

## 1. Introduction

The World Health Organization (WHO) announced a novel coronavirus disease (COVID-19) as a pandemic on 11 March 2020 due to its speed and global transmission [1]. Since then, we are severely affected by the nations with vast numbers of deaths; it was hard to predict such a colossal pandemic with terrifying consequences with a significant influence regardless of any countries, developed, developing or least developed. By 22 April 2020, WHO has reported 2,471,136 confirmed cases with 169,006 deaths worldwide [1].

A global public health problem with a high rate spread and transmission, coronavirus outbreak has become the most talked-about matter throughout the world. Health care providers are at the forefront of all others, and the current crisis is such a difficult time for them. Non-urgent elective surgeries have been cancelled or postponed to free up beds for coronavirus critically ill patients, to allow the best use of medical resources for both the patients and the health care providers, and to reduce the contamination risk of healthy people.

Although elective surgeries are limited during the COVID-19 pandemic, situations requiring an urgent gynaecological or obstetric surgical approach must still be performed. It is always essential to apply the most appropriate, personalized treatment modality to patients and to carry out the appropriate and safe treatment by considering safety for both patients and health care providers, adhering to the principle of not harming the highest perception. The routes chosen to perform the surgery either by open or by minimally invasive (laparoscopy, robotics or vaginally) techniques, it is vital to follow patient management algorithms prepared within the evidence during COVID-19 pandemic. At this point, concerns regarding surgical safety and the risk of viral transmission during surgery are of great importance. For this purpose, we prepared a review that summarizes the concepts that should be considered about laparoscopic gynaecological surgery during the COVID-19 pandemic in the light of current literature. 

## 2. Surgical safety and advantages of laparoscopy during the COVID-19 pandemic

Since January, in the three months, changing in the actual policies and getting out of the routine, the decision of the route of surgery either by traditional open surgery or laparoscopic surgery is being discussed and stated regarding surgical safety concerns [2]. The first decision arises from the thought if the patient’s situation needs an urgent intervention or not. The second is that if the patient needs urgent surgery, what will be the route, laparoscopy or laparotomy?

According to our traditional knowledge, the use of laparoscopy offers many benefits to patients such as shorter recovery time and hospital stay, lower risk of post-surgical complications, reduced risk of pain and pain medication, less bleeding and risk of haemorrhage during the operation [3]. If looked at the point of the surgeon and surgical staff, laparoscopy offers less or no spillage of fluids and tissues, and also provides more distance between each surgeon and the patient. Sure, the risks and benefits should be weighed, and the most appropriate decision should be taken after the patient, and the current situation are evaluated in detail. 

Recently, international societies have made recommendations based on published reasonable data and expert opinion about laparoscopic surgery during coronavirus outbreak. The American Association of Gynecologic Laparoscopists (AAGL), along with other societies as the American College of Obstetricians and Gynecologists (ACOG), the American Society of Reproductive Medicine (ASRM), and others released the Joint Statement regarding the suspension of elective surgical care during the first phase of the pandemic [4]. The Royal College of Obstetricians and Gynaecologists (RCOG) with the British Society for Gynaecological Endoscopists (BSGE) stated that laparoscopic approaches should be performed when feasible in preference to laparotomy in guidance with the safety of surgeons and patients. The main reason for the preference of laparoscopy is that of its benefits not only for the patients but also for the better use of hospital resources at these unusual times [5]. The European Society for Gynaecological Endoscopy (ESGE) also declared that elective surgeries for benign conditions should be delayed, and if possible, alternate medical treatments should be considered. If a gynaecological emergency takes place, laparoscopic surgery should be advantageous, weighing all the consequences [6]. In light of all limited data, it may be hard to find the right way to act. At the same time, some scientists advocate the superiority and the high preferability of minimally invasive surgery in all patients [7], some scientists defend the triage, testing and protection, and minimize and delay the surgical decision on COVID-19 (+) patients [8]. The same authors argue that if the patient needs urgent surgery, and not have enough time to test the patient, laparotomy should be performed to minimize the risks. In this instance, the best way to act is as Brown explains: “all laparoscopy” or “all laparotomy” is not appropriate, algorithms based on risk reduction need to be attributed to the situation you are in [2]. For all that, deciding the route of the operation may differ upon hospital settings, the condition of the patients and the availability of screening. Recommendations based on current evidence and expert opinion are given as follows.

### 2.1. General comments

**Figure 1:**
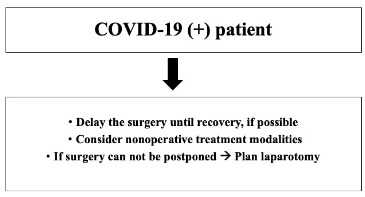
Recommendations in a COVID-19 (+) patient.

**Figure 2:**
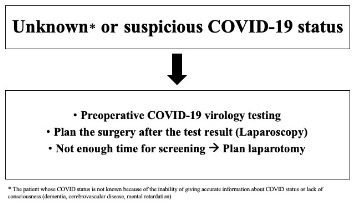
Recommendations in a patient with unknown or suspicious status.

**Figure 3:**
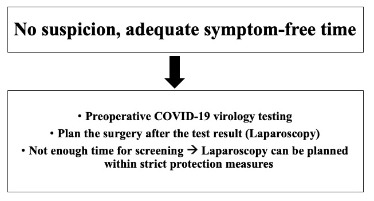
Recommendations in a patient with no suspicious status.

At admission, all patients requiring urgent surgery should be assessed for potential SARS-CoV-2 infection including general screening questions as having contacts with a COVID-19 (+) patient within the last 14 days or having any symptoms or not [9]. Informed consent concerning the probability of COVID-19 exposure and potential consequences should be discussed with the patient [10]. Our management protocols in patients with different COVID-19 status are stated below (Figures 1–3).

*COVID-19 (+) patients*: Gynecologic procedures, for which a delay will not negatively affect patient health and safety, should be delayed [4]. If there is no life-threatening condition in the COVID-19 (+) patient requiring surgery, the surgical treatment should be postponed until full recovery, nonoperative treatment modalities may be implemented when possible [11]. Although different attitudes between laparoscopy and laparotomy are being discussed in COVID-19 (+) patients [7,8], we recommend laparotomy at our institution if surgery cannot be postponed. If the decision is in the direction of laparotomy, minilaparotomy should be performed if possible (Figure 1).

*Unknown or Suspicious COVID-19 status*: If the patient who needs urgent surgery and whose COVID-19 status is not known because of the inability of giving accurate information about COVID status or lack of consciousness (dementia, cerebrovascular disease, mental retardation etc.), it is appropriate to perform preoperative COVID-19 virology screening, and plan the surgery after the result is obtained. If there is not enough time for preoperative COVID-19 screening, urgent surgery should be performed by laparotomy (Figure 2). 

*No suspicion, adequate symptom-free time: *If there is not enough time for preoperative COVID-19 screening, the surgery can be performed by laparoscopy within taking strict protection measures. It should not be forgotten to take every precaution before every surgical procedure irrespective of the testing results of the patients (Figure 3).

### 2.2. Operating room (OR) environment

An OR with a negative pressure environment is defined ideal for the reduction of the dissemination of the virus [12]. If available, an alternative OR separated from main ORs only for COVID-19 patients would be ideal to avoid contamination of other ORs and patients. It is stated that a high frequency of filtered air exchange helps for the reduction of the viral load within the OR [13]. Detailed hospital guidelines, including workflow definitions and new OR rules, appropriate donning, and doffing procedures, should be defined to standardize the procedures and coordination of all surgical staff.

### 2.3. Personal protective equipment (PPE)

In patients with confirmed or suspected COVID-19 infection, all surgical team members are required to wear PPE. The WHO recommends that FFP2/3 and N95 masks can be for up to 4 h [14]. When aerosol-generating procedures are performed, and until air exchanges have reduced the virus after the procedure, airborne precaution PPE is recommended and should be worn by all surgical staff within the OR during all operations, whether by laparoscopy or laparotomy [5,15]. Level III protection of PPE includes face shields, FFP2/3 or N95 filtered masks, fluid-resistant gowns, disposable gloves, disposable eye protection [13]. Standard infection controls should be already applied, but the types of transmission and the protection required to handle that of transmission should be also known exactly (Table 1) [15]. 

**Table 1:** Table 1. Routes of transmission and PPE use [15].

Route of transmission	When to use	PPE
Contact precaution	> 2 m away from the patient	Gloves Apron
Droplet precaution	Within 2 m of the patient	Gloves Apron Fluid resistant surgical mask +/- Eye protection (risk assess)
Airborne precaution	Aerosol generating procedure	Gloves Fluid proof long sleeved gown Eye protection (Protective glasses) FFP3 mask

### 2.4. Viral transmission 

Laparoscopic surgery is a surgical method performed by forming the pneumoperitoneum by inflating the abdominal compartment with CO2. Theoretically, it is possible to aerosolize viral particles and contaminate the operating room environment by using the gas supplied into the abdomen and using electrosurgery and ultrasonic devices during the operation; that means laparoscopic operations are aerosol-generating procedures (AGP). COVID-19 virus (SARS-CoV-2) is a respiratory agent transmitted via respiratory droplets [7]. The mechanisms are thought to be in three ways: (a) directly by *droplets *from human to human via someone’s nose, mouth, or eyes or (b) smaller but much more numerous particles called “*aerosol particles*” or (c) from *contaminated surfaces* with larger droplets that spread onto the surfaces from an infected person’s secretions [16]. Sites of deposition in the recipient differ between inhaled droplets and aerosol particles; while the bigger ones localize in upper regions of the respiratory tract, the inhaled aerosol particles penetrate deeper into the lungs [17]. In laparoscopy, aerosolized particles are produced mainly by electrosurgical smoke via energy devices. In contrast, particles with the smallest mean size (<0.1 µm) were formed by electrocautery; the largest ones sized 0.35–6.5 µm were shown to be generated by use of the ultrasonic scalpels [18]. The role of CO2 in aerosol formation remains unclear [16]. Based on our previous information about the presence of Hepatitis B [19], human immunodeficiency viruses (HIV) [20], and similar respiratory viruses as influenza or coronaviruses [Severe Acute Respiratory Syndrome (SARS) or Middle East Respiratory Syndrome (MERS)] in surgical plume during laparoscopy, to date, no study presents the ability of viral transmission by laparoscopy [16,21]. Besides, SARS-CoV-2 has not been detected in AGPs yet, but the virus has been detected in blood in 1% of cases and stool specimens in 29% of cases [21,22]. Although the virus has not been reported in the genital tract yet, COVID-19 seems to be very contagious, and what we do not know is much more than we know about the virus at present. At this point, the most critical aspect of the event is to protect ourselves from potentially dangerous biological materials and minimize the risks we face as much as possible with the use of protective maneuvers and techniques. 

In laparoscopy, to minimize the use of electrosurgical procedures, especially laser tissue ablation, monopolar electrosurgery, ultrasonic scalpels and advanced bipolar devices, may reduce the probable risk of viral emission [10]. The sudden opening of the trocars causing a chimney effect (a jet stream through the trocars) [18] towards the surgical team or specimen extraction with free gas leakage through the abdominal and vaginal incisions or uncontrolled replacement of laparoscopic instruments at the end of the operation may expose the medical team to these aerosolized viral particles. High-efficiency particulate air (HEPA) filters and Ultra-low particulate air (ULPA) filters can remove >99% of airborne particles [10], and use of a closed smoke evacuation/filtration system with ULPA capability is recommended if available [11]. Using closed smoke evacuation filtering devices may protect the team against the unknown risk of COVID-19 transmission [23].

### 2.5. Anaesthesia

Regional anaesthesia options should be discussed with anaesthesiologists in vaginal and open surgeries to avoid aerosol-generating procedures, including intubation and extubation [11]. If general anaesthesia and intubation are required, COVID-19 guidance for anaesthesia and all team members should be considered [24].

### 2.6. During the operation

It is vital to operate in the shortest time by an experienced laparoscopic surgeon. To minimize the number of staff in the operating room is highly recommended to provide safety. As the knowledge of the transmission ability of aerosolized virus during laparoscopy is lacking, all staff in the OR should use PPE. To use the trocar sites effectively for safer and faster surgery, the size and number of the incisions for trocars should be appropriate enough, not too small or big with the lowest numbers. Intraabdominal pressure should be as low as possible (10–11mmHg) with available Trendelenburg position to establish the pneumoperitoneum [5]. The taps of the trocars should be closed before insertion and removal processes to avoid leakage of not only the gas but also the body fluids; it should always be remembered that to minimize the instrument exchange leads to minimizing leakage [13]. It is recommended to lower the electrocautery power settings as possible; caution with ultrasonic devices, monopolar electrocautery and avoidance of prolonged desiccation is considerable. While removing specimens such as in ectopic pregnancy, it is rational to deflate the abdomen with a suction device before removing the bag from the abdomen [6]. Surgeries that carry a high risk of bowel involvement as tuboovarian abscess, known pelvic adhesions should be performed by laparotomy [5,7,13] (Table 2). 

**Table 2:** Specific recommendations for laparoscopy.

1. Set the intraabdominal pressure as low as possible (10–11 mmHg). 2. Close the taps of the trocars before insertion and during the operation. 3. Pay maximum attention to port sites; (a) a minimum number of incisions, (b) minimum size of incisions, (c) minimum exchange of the instruments. 4. Minimize the use of energy devices, lower the electrocautery power settings as possible; (a) avoid using ultrasonic devices, (b) avoid prolonged desiccation. 5. Consider using vacuum suction devices, a closed-circuit smoke evacuation device with a HEPA filter or a ULPA filter if possible. 6. Make sure that the taps of the trocars are closed all the time unless evacuation is achieved. 7. Make sure that the pneumoperitoneum and smoke is safely evacuated before specimen extraction, trocar removal, closure of the incisions or conversion to laparotomy.

HEPA: High-efficiency particulate air; ULPA: Ultra-low particulate air.

## 3. Conclusion

On the review of the present data about the viral transmission of COVID-19, performing surgery in any way does not differ in terms of open surgery or laparoscopy. However, it should be remembered that minimally invasive approaches shorten the recovery and hospitalization period of the patients. All precautions should be taken into consideration, including both protective equipment and recommendations about any type of surgery that may have a risk of aerosolization. Evidence-based data and expert opinions renewing every day about the issue will address the pros and cons of the way of surgery during COVID-19 in the coming period.
